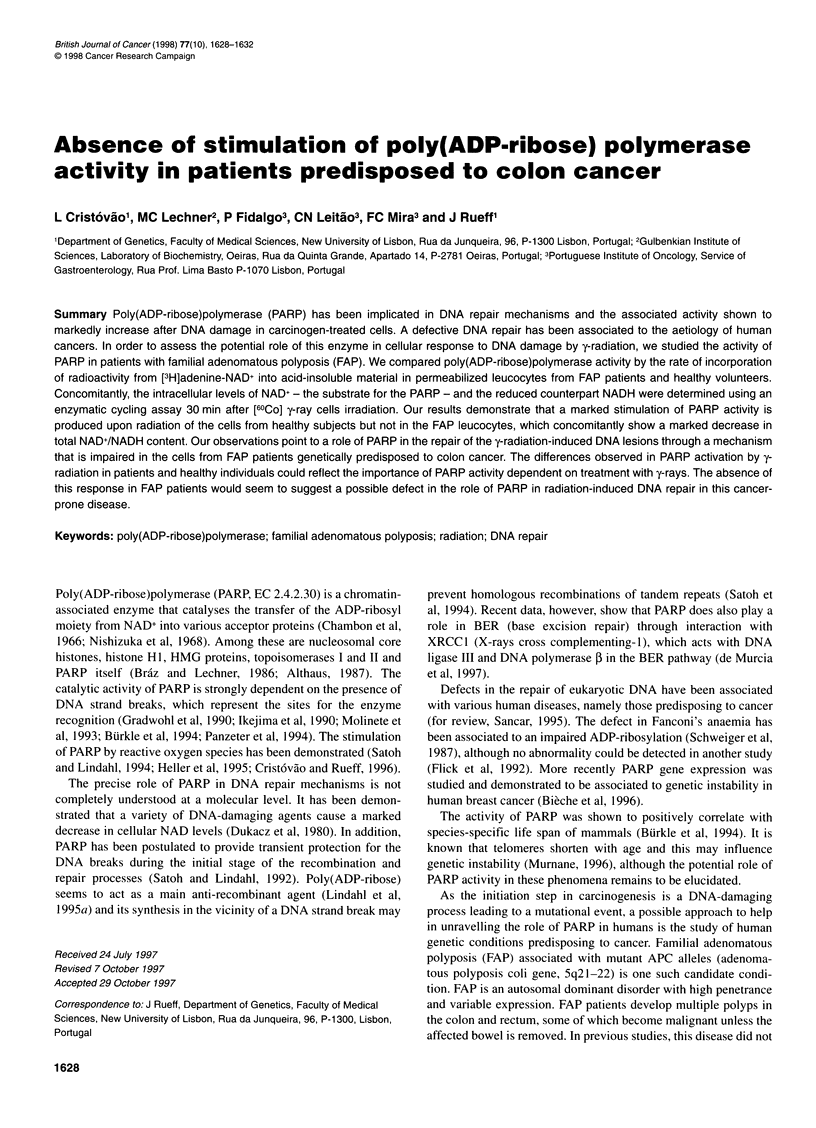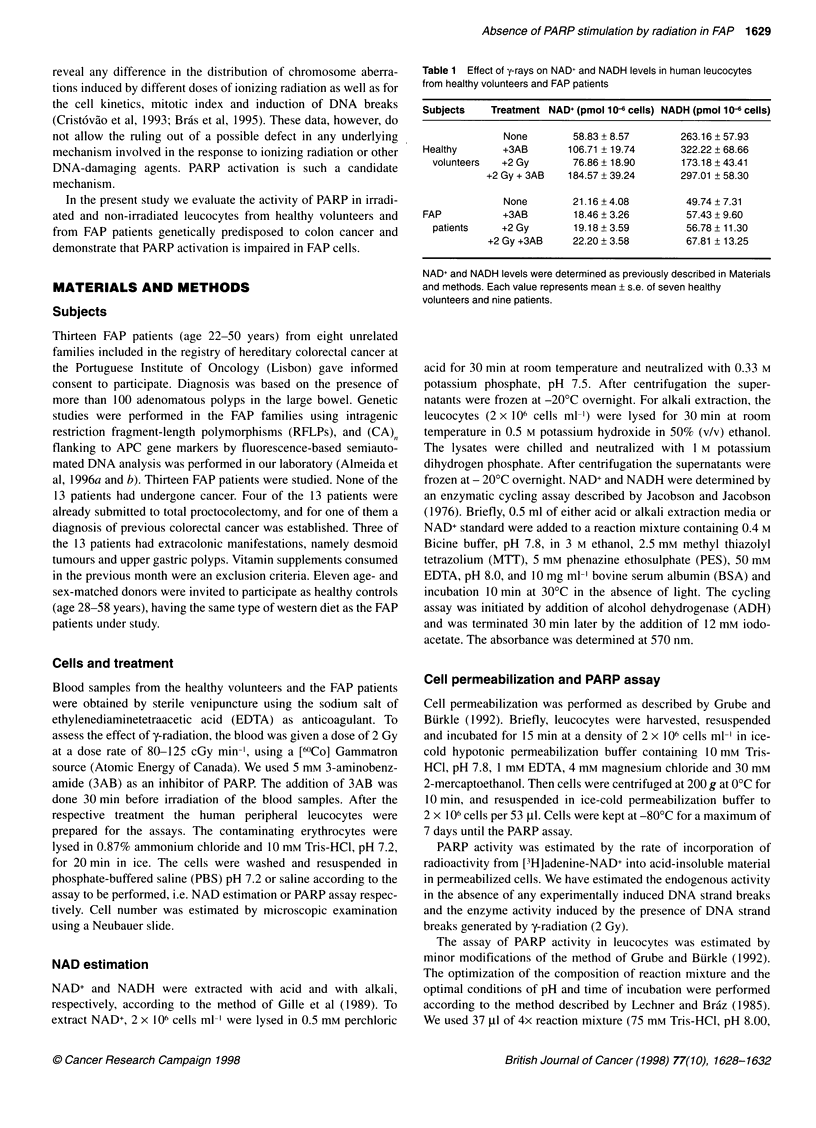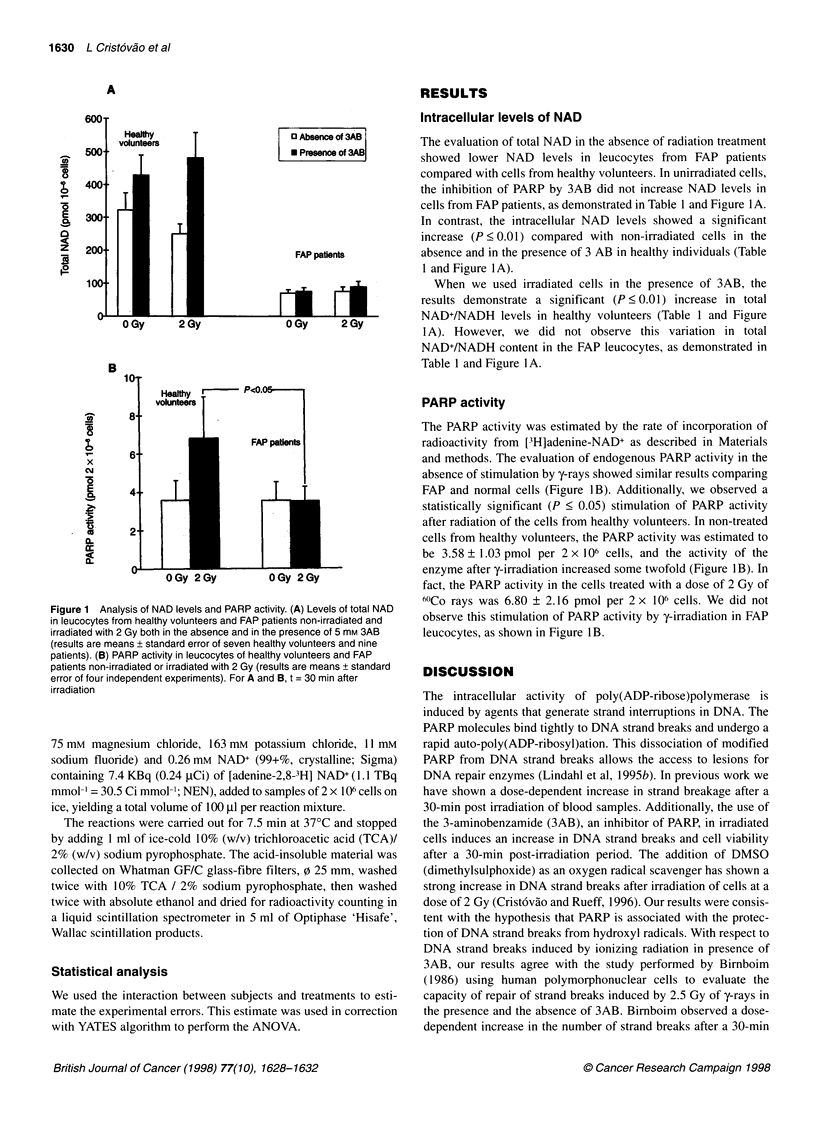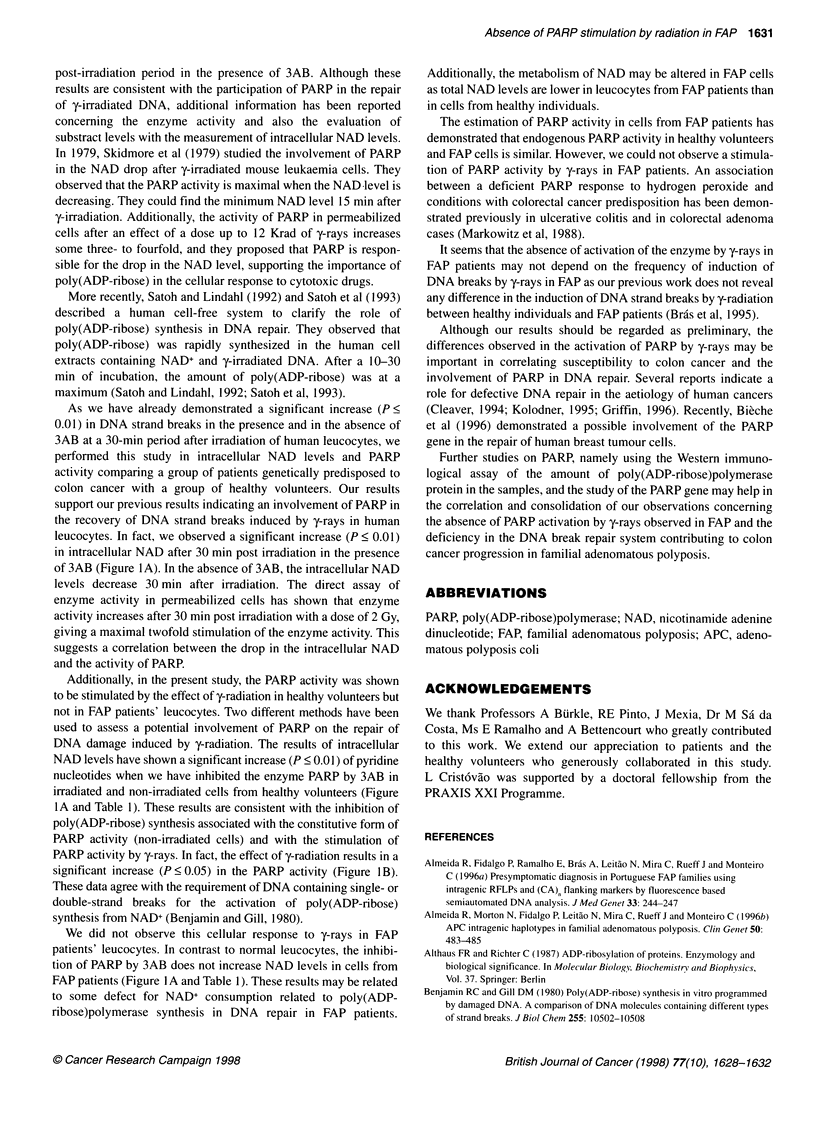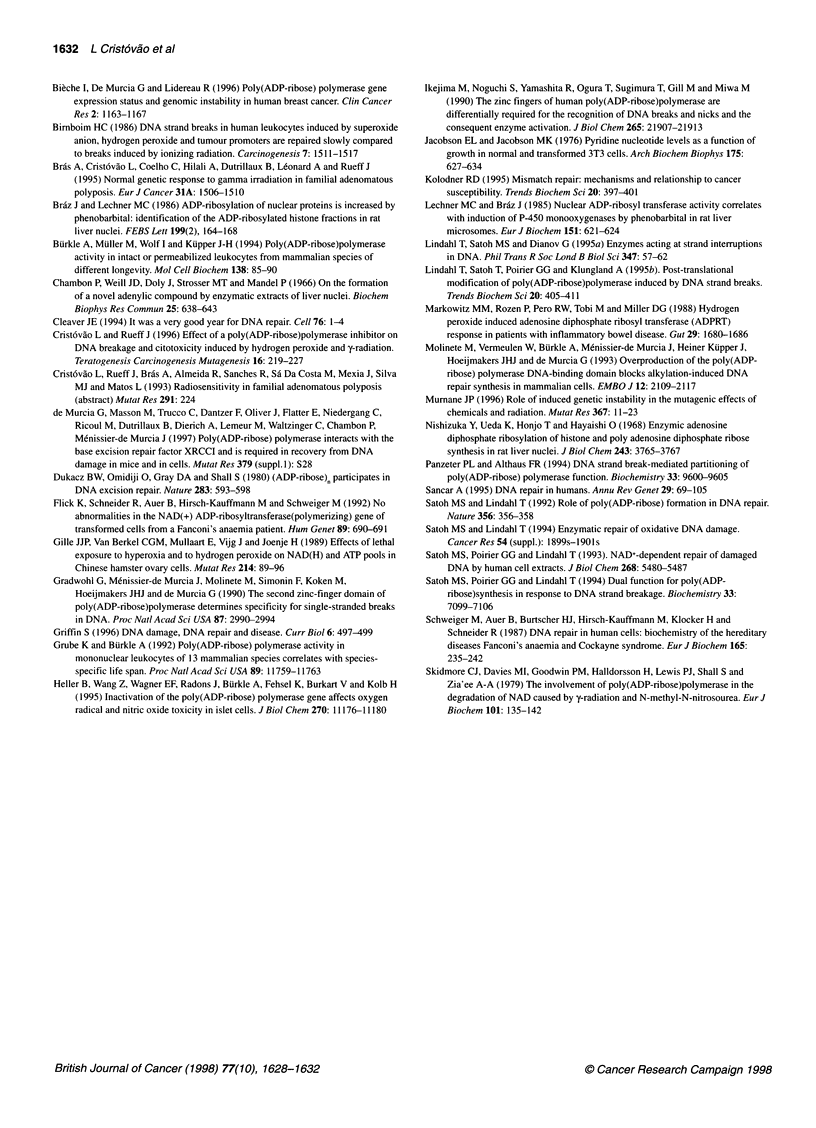# Absence of stimulation of poly(ADP-ribose) polymerase activity in patients predisposed to colon cancer.

**DOI:** 10.1038/bjc.1998.266

**Published:** 1998-05

**Authors:** L. CristÃ³vÃ£o, M. C. Lechner, P. Fidalgo, C. N. LeitÃ£o, F. C. Mira, J. Rueff

**Affiliations:** Department of Genetics, Faculty of Medical Sciences, New University of Lisbon, Portugal.

## Abstract

Poly(ADP-ribose)polymerase (PARP) has been implicated in DNA repair mechanisms and the associated activity shown to markedly increase after DNA damage in carcinogen-treated cells. A defective DNA repair has been associated to the aetiology of human cancers. In order to assess the potential role of this enzyme in cellular response to DNA damage by gamma-radiation, we studied the activity of PARP in patients with familial adenomatous polyposis (FAP). We compared poly(ADP-ribose)polymerase activity by the rate of incorporation of radioactivity from [3H]adenine-NAD+ into acid-insoluble material in permeabilized leucocytes from FAP patients and healthy volunteers. Concomitantly, the intracellular levels of NAD+--the substrate for the PARP--and the reduced counterpart NADH were determined using an enzymatic cycling assay 30 min after [60Co] gamma-ray cells irradiation. Our results demonstrate that a marked stimulation of PARP activity is produced upon radiation of the cells from healthy subjects but not in the FAP leucocytes, which concomitantly show a marked decrease in total NAD-/NADH content. Our observations point to a role of PARP in the repair of the gamma-radiation-induced DNA lesions through a mechanism that is impaired in the cells from FAP patients genetically predisposed to colon cancer. The differences observed in PARP activation by gamma-radiation in patients and healthy individuals could reflect the importance of PARP activity dependent on treatment with gamma-rays. The absence of this response in FAP patients would seem to suggest a possible defect in the role of PARP in radiation-induced DNA repair in this cancer-prone disease.


British Journal of Cancer (1998) 77(10), 1628–1632
© 1998 Cancer Research Campaign

### Absence of stimulation of poly(ADP-ribose) polymerase activity in patients predisposed to colon cancer

**L Cristóvão[1], MC Lechner[2], P Fidalgo[3], CN Leitão[3], FC Mira[3] and J Rueff[1]**

[1]Department of Genetics, Faculty of Medical Sciences, New University of Lisbon, Rua da Junqueira, 96, P-1300 Lisbon, Portugal; [2]Gulbenkian Institute of Sciences, Laboratory of Biochemistry, Oeiras, Rua da Quinta Grande, Apartado 14, P-2781 Oeiras, Portugal; [3]Portuguese Institute of Oncology, Service of Gastroenterology, Rua Prof. Lima Basto P-1070 Lisbon, Portugal

**Summary** Poly(ADP-ribose)polymerase (PARP) has been implicated in DNA repair mechanisms and the associated activity shown to markedly increase after DNA damage in carcinogen-treated cells. A defective DNA repair has been associated to the aetiology of human cancers. In order to assess the potential role of this enzyme in cellular response to DNA damage by γ-radiation, we studied the activity of PARP in patients with familial adenomatous polyposis (FAP). We compared poly(ADP-ribose)polymerase activity by the rate of incorporation of radioactivity from [$^3$H]adenine-NAD$^+$ into acid-insoluble material in permeabilized leucocytes from FAP patients and healthy volunteers. Concomitantly, the intracellular levels of NAD$^+$ – the substrate for the PARP – and the reduced counterpart NADH were determined using an enzymatic cycling assay 30 min after [$^{60}$Co] γ-ray cells irradiation. Our results demonstrate that a marked stimulation of PARP activity is produced upon radiation of the cells from healthy subjects but not in the FAP leucocytes, which concomitantly show a marked decrease in total NAD$^+$/NADH content. Our observations point to a role of PARP in the repair of the γ-radiation-induced DNA lesions through a mechanism that is impaired in the cells from FAP patients genetically predisposed to colon cancer. The differences observed in PARP activation by γ-radiation in patients and healthy individuals could reflect the importance of PARP activity dependent on treatment with γ-rays. The absence of this response in FAP patients would seem to suggest a possible defect in the role of PARP in radiation-induced DNA repair in this cancer-prone disease.

**Keywords:** poly(ADP-ribose)polymerase; familial adenomatous polyposis; radiation; DNA repair

Poly(ADP-ribose)polymerase (PARP, EC 2.4.2.30) is a chromatin-associated enzyme that catalyses the transfer of the ADP-ribosyl moiety from NAD$^+$ into various acceptor proteins (Chambon et al, 1966; Nishizuka et al, 1968). Among these are nucleosomal core histones, histone H1, HMG proteins, topoisomerases I and II and PARP itself (Bráz and Lechner, 1986; Althaus, 1987). The catalytic activity of PARP is strongly dependent on the presence of DNA strand breaks, which represent the sites for the enzyme recognition (Gradwohl et al, 1990; Ikejima et al, 1990; Molinete et al, 1993; Bürkle et al, 1994; Panzeter et al, 1994). The stimulation of PARP by reactive oxygen species has been demonstrated (Satoh and Lindahl, 1994; Heller et al, 1995; Cristóvão and Rueff, 1996).

The precise role of PARP in DNA repair mechanisms is not completely understood at a molecular level. It has been demonstrated that a variety of DNA-damaging agents cause a marked decrease in cellular NAD levels (Dukacz et al, 1980). In addition, PARP has been postulated to provide transient protection for the DNA breaks during the initial stage of the recombination and repair processes (Satoh and Lindahl, 1992). Poly(ADP-ribose) seems to act as a main anti-recombinant agent (Lindahl et al, 1995*a*) and its synthesis in the vicinity of a DNA strand break may prevent homologous recombinations of tandem repeats (Satoh et al, 1994). Recent data, however, show that PARP does also play a role in BER (base excision repair) through interaction with XRCC1 (X-rays cross complementing-1), which acts with DNA ligase III and DNA polymerase β in the BER pathway (de Murcia et al, 1997).

Defects in the repair of eukaryotic DNA have been associated with various human diseases, namely those predisposing to cancer (for review, Sancar, 1995). The defect in Fanconi's anaemia has been associated to an impaired ADP-ribosylation (Schweiger et al, 1987), although no abnormality could be detected in another study (Flick et al, 1992). More recently PARP gene expression was studied and demonstrated to be associated to genetic instability in human breast cancer (Bièche et al, 1996).

The activity of PARP was shown to positively correlate with species-specific life span of mammals (Bürkle et al, 1994). It is known that telomeres shorten with age and this may influence genetic instability (Murnane, 1996), although the potential role of PARP activity in these phenomena remains to be elucidated.

As the initiation step in carcinogenesis is a DNA-damaging process leading to a mutational event, a possible approach to help in unravelling the role of PARP in humans is the study of human genetic conditions predisposing to cancer. Familial adenomatous polyposis (FAP) associated with mutant APC alleles (adenomatous polyposis coli gene, 5q21–22) is one such candidate condition. FAP is an autosomal dominant disorder with high penetrance and variable expression. FAP patients develop multiple polyps in the colon and rectum, some of which become malignant unless the affected bowel is removed. In previous studies, this disease did not

*Received 24 July 1997*
*Revised 7 October 1997*
*Accepted 29 October 1997*

*Correspondence to:* J Rueff, Department of Genetics, Faculty of Medical Sciences, New University of Lisbon, Rua da Junqueira, 96, P-1300, Lisbon, Portugal reveal any difference in the distribution of chromosome aberrations induced by different doses of ionizing radiation as well as for the cell kinetics, mitotic index and induction of DNA breaks (Cristóvão et al, 1993; Brás et al, 1995). These data, however, do not allow the ruling out of a possible defect in any underlying mechanism involved in the response to ionizing radiation or other DNA-damaging agents. PARP activation is such a candidate mechanism.

In the present study we evaluate the activity of PARP in irradiated and non-irradiated leucocytes from healthy volunteers and from FAP patients genetically predisposed to colon cancer and demonstrate that PARP activation is impaired in FAP cells.

**Table 1** Effect of γ-rays on $NAD^+$ and NADH levels in human leucocytes from healthy volunteers and FAP patients

| Subjects | Treatment | $NAD^+$ (pmol $10^{-6}$ cells) | NADH (pmol $10^{-6}$ cells) |
|---|---|---|---|
| | None | 58.83 ± 8.57 | 263.16 ± 57.93 |
| Healthy | +3AB | 106.71 ± 19.74 | 322.22 ± 68.66 |
| volunteers | +2 Gy | 76.86 ± 18.90 | 173.18 ± 43.41 |
| | +2 Gy + 3AB | 184.57 ± 39.24 | 297.01 ± 58.30 |
| | None | 21.16 ± 4.08 | 49.74 ± 7.31 |
| FAP | +3AB | 18.46 ± 3.26 | 57.43 ± 9.60 |
| patients | +2 Gy | 19.18 ± 3.59 | 56.78 ± 11.30 |
| | +2 Gy +3AB | 22.20 ± 3.58 | 67.81 ± 13.25 |

$NAD^+$ and NADH levels were determined as previously described in Materials and methods. Each value represents mean ± s.e. of seven healthy volunteers and nine patients.

### MATERIALS AND METHODS

### Subjects

Thirteen FAP patients (age 22–50 years) from eight unrelated families included in the registry of hereditary colorectal cancer at the Portuguese Institute of Oncology (Lisbon) gave informed consent to participate. Diagnosis was based on the presence of more than 100 adenomatous polyps in the large bowel. Genetic studies were performed in the FAP families using intragenic restriction fragment-length polymorphisms (RFLPs), and $(CA)_n$ flanking to APC gene markers by fluorescence-based semiautomated DNA analysis was performed in our laboratory (Almeida et al, 1996*a* and *b*). Thirteen FAP patients were studied. None of the 13 patients had undergone cancer. Four of the 13 patients were already submitted to total proctocolectomy, and for one of them a diagnosis of previous colorectal cancer was established. Three of the 13 patients had extracolonic manifestations, namely desmoid tumours and upper gastric polyps. Vitamin supplements consumed in the previous month were an exclusion criteria. Eleven age- and sex-matched donors were invited to participate as healthy controls (age 28–58 years), having the same type of western diet as the FAP patients under study.

### Cells and treatment

Blood samples from the healthy volunteers and the FAP patients were obtained by sterile venipuncture using the sodium salt of ethylenediaminetetraacetic acid (EDTA) as anticoagulant. To assess the effect of γ-radiation, the blood was given a dose of 2 Gy at a dose rate of 80–125 cGy $min^{-1}$, using a [$^{60}$Co] Gammatron source (Atomic Energy of Canada). We used 5 mM 3-aminobenzamide (3AB) as an inhibitor of PARP. The addition of 3AB was done 30 min before irradiation of the blood samples. After the respective treatment the human peripheral leucocytes were prepared for the assays. The contaminating erythrocytes were lysed in 0.87% ammonium chloride and 10 mM Tris-HCl, pH 7.2, for 20 min in ice. The cells were washed and resuspended in phosphate-buffered saline (PBS) pH 7.2 or saline according to the assay to be performed, i.e. NAD estimation or PARP assay respectively. Cell number was estimated by microscopic examination using a Neubauer slide.

### NAD estimation

$NAD^+$ and NADH were extracted with acid and with alkali, respectively, according to the method of Gille et al (1989). To extract $NAD^+$, $2 \times 10^6$ cells $ml^{-1}$ were lysed in 0.5 mM perchloric acid for 30 min at room temperature and neutralized with 0.33 M potassium phosphate, pH 7.5. After centrifugation the supernatants were frozen at –20°C overnight. For alkali extraction, the leucocytes ($2 \times 10^6$ cells $ml^{-1}$) were lysed for 30 min at room temperature in 0.5 M potassium hydroxide in 50% (v/v) ethanol. The lysates were chilled and neutralized with 1 M potassium dihydrogen phosphate. After centrifugation the supernatants were frozen at – 20°C overnight. $NAD^+$ and NADH were determined by an enzymatic cycling assay described by Jacobson and Jacobson (1976). Briefly, 0.5 ml of either acid or alkali extraction media or $NAD^+$ standard were added to a reaction mixture containing 0.4 M Bicine buffer, pH 7.8, in 3 M ethanol, 2.5 mM methyl thiazolyl tetrazolium (MTT), 5 mM phenazine ethosulphate (PES), 50 mM EDTA, pH 8.0, and 10 mg $ml^{-1}$ bovine serum albumin (BSA) and incubation 10 min at 30°C in the absence of light. The cycling assay was initiated by addition of alcohol dehydrogenase (ADH) and was terminated 30 min later by the addition of 12 mM iodoacetate. The absorbance was determined at 570 nm.

### Cell permeabilization and PARP assay

Cell permeabilization was performed as described by Grube and Bürkle (1992). Briefly, leucocytes were harvested, resuspended and incubated for 15 min at a density of $2 \times 10^6$ cells $ml^{-1}$ in ice-cold hypotonic permeabilization buffer containing 10 mM Tris-HCl, pH 7.8, 1 mM EDTA, 4 mM magnesium chloride and 30 mM 2-mercaptoethanol. Then cells were centrifuged at 200 ***g*** at 0°C for 10 min, and resuspended in ice-cold permeabilization buffer to $2 \times 10^6$ cells per 53 μl. Cells were kept at –80°C for a maximum of 7 days until the PARP assay.

PARP activity was estimated by the rate of incorporation of radioactivity from [$^3$H]adenine-$NAD^+$ into acid-insoluble material in permeabilized cells. We have estimated the endogenous activity in the absence of any experimentally induced DNA strand breaks and the enzyme activity induced by the presence of DNA strand breaks generated by γ-radiation (2 Gy).

The assay of PARP activity in leucocytes was estimated by minor modifications of the method of Grube and Bürkle (1992). The optimization of the composition of reaction mixture and the optimal conditions of pH and time of incubation were performed according to the method described by Lechner and Bráz (1985). We used 37 μl of 4× reaction mixture (75 mM Tris-HCl, pH 8.00,

© Cancer Research Campaign 1998

**Figure 1** Analysis of NAD levels and PARP activity. (**A**) Levels of total NAD in leucocytes from healthy volunteers and FAP patients non-irradiated and irradiated with 2 Gy both in the absence and in the presence of 5 mM 3AB (results are means ± standard error of seven healthy volunteers and nine patients). (**B**) PARP activity in leucocytes of healthy volunteers and FAP patients non-irradiated or irradiated with 2 Gy (results are means ± standard error of four independent experiments). For **A** and **B**, t = 30 min after irradiation

75 mM magnesium chloride, 163 mM potassium chloride, 11 mM sodium fluoride) and 0.26 mM $NAD^+$ (99+%, crystalline; Sigma) containing 7.4 KBq (0.24 µCi) of [adenine-2,8-$^3$H] $NAD^+$ (1.1 TBq $mmol^{-1}$ = 30.5 Ci $mmol^{-1}$; NEN), added to samples of $2 \times 10^6$ cells on ice, yielding a total volume of 100 µl per reaction mixture.

The reactions were carried out for 7.5 min at 37°C and stopped by adding 1 ml of ice-cold 10% (w/v) trichloroacetic acid (TCA)/ 2% (w/v) sodium pyrophosphate. The acid-insoluble material was collected on Whatman GF/C glass-fibre filters, ø 25 mm, washed twice with 10% TCA / 2% sodium pyrophosphate, then washed twice with absolute ethanol and dried for radioactivity counting in a liquid scintillation spectrometer in 5 ml of Optiphase 'Hisafe', Wallac scintillation products.

### Statistical analysis

We used the interaction between subjects and treatments to estimate the experimental errors. This estimate was used in correction with YATES algorithm to perform the ANOVA.

### RESULTS

### Intracellular levels of NAD

The evaluation of total NAD in the absence of radiation treatment showed lower NAD levels in leucocytes from FAP patients compared with cells from healthy volunteers. In unirradiated cells, the inhibition of PARP by 3AB did not increase NAD levels in cells from FAP patients, as demonstrated in Table 1 and Figure 1A. In contrast, the intracellular NAD levels showed a significant increase ($P \leq 0.01$) compared with non-irradiated cells in the absence and in the presence of 3 AB in healthy individuals (Table 1 and Figure 1A).

When we used irradiated cells in the presence of 3AB, the results demonstrate a significant ($P \leq 0.01$) increase in total $NAD^+$/NADH levels in healthy volunteers (Table 1 and Figure 1A). However, we did not observe this variation in total $NAD^+$/NADH content in the FAP leucocytes, as demonstrated in Table 1 and Figure 1A.

### PARP activity

The PARP activity was estimated by the rate of incorporation of radioactivity from [$^3$H]adenine-$NAD^+$ as described in Materials and methods. The evaluation of endogenous PARP activity in the absence of stimulation by γ-rays showed similar results comparing FAP and normal cells (Figure 1B). Additionally, we observed a statistically significant ($P \leq 0.05$) stimulation of PARP activity after radiation of the cells from healthy volunteers. In non-treated cells from healthy volunteers, the PARP activity was estimated to be 3.58 ± 1.03 pmol per $2 \times 10^6$ cells, and the activity of the enzyme after γ-irradiation increased some twofold (Figure 1B). In fact, the PARP activity in the cells treated with a dose of 2 Gy of $^{60}$Co rays was 6.80 ± 2.16 pmol per $2 \times 10^6$ cells. We did not observe this stimulation of PARP activity by γ-irradiation in FAP leucocytes, as shown in Figure 1B.

### DISCUSSION

The intracellular activity of poly(ADP-ribose)polymerase is induced by agents that generate strand interruptions in DNA. The PARP molecules bind tightly to DNA strand breaks and undergo a rapid auto-poly(ADP-ribosyl)ation. This dissociation of modified PARP from DNA strand breaks allows the access to lesions for DNA repair enzymes (Lindahl et al, 1995*b*). In previous work we have shown a dose-dependent increase in strand breakage after a 30-min post irradiation of blood samples. Additionally, the use of the 3-aminobenzamide (3AB), an inhibitor of PARP, in irradiated cells induces an increase in DNA strand breaks and cell viability after a 30-min post-irradiation period. The addition of DMSO (dimethylsulphoxide) as an oxygen radical scavenger has shown a strong increase in DNA strand breaks after irradiation of cells at a dose of 2 Gy (Cristóvão and Rueff, 1996). Our results were consistent with the hypothesis that PARP is associated with the protection of DNA strand breaks from hydroxyl radicals. With respect to DNA strand breaks induced by ionizing radiation in presence of 3AB, our results agree with the study performed by Birnboim (1986) using human polymorphonuclear cells to evaluate the capacity of repair of strand breaks induced by 2.5 Gy of γ-rays in the presence and the absence of 3AB. Birnboim observed a dose-dependent increase in the number of strand breaks after a 30-min

 *© Cancer Research Campaign 1998*

post-irradiation period in the presence of 3AB. Although these results are consistent with the participation of PARP in the repair of γ-irradiated DNA, additional information has been reported concerning the enzyme activity and also the evaluation of substract levels with the measurement of intracellular NAD levels. In 1979, Skidmore et al (1979) studied the involvement of PARP in the NAD drop after γ-irradiated mouse leukaemia cells. They observed that the PARP activity is maximal when the NAD level is decreasing. They could find the minimum NAD level 15 min after γ-irradiation. Additionally, the activity of PARP in permeabilized cells after an effect of a dose up to 12 Krad of γ-rays increases some three- to fourfold, and they proposed that PARP is responsible for the drop in the NAD level, supporting the importance of poly(ADP-ribose) in the cellular response to cytotoxic drugs.

More recently, Satoh and Lindahl (1992) and Satoh et al (1993) described a human cell-free system to clarify the role of poly(ADP-ribose) synthesis in DNA repair. They observed that poly(ADP-ribose) was rapidly synthesized in the human cell extracts containing $NAD^+$ and γ-irradiated DNA. After a 10–30 min of incubation, the amount of poly(ADP-ribose) was at a maximum (Satoh and Lindahl, 1992; Satoh et al, 1993).

As we have already demonstrated a significant increase ($P \leq 0.01$) in DNA strand breaks in the presence and in the absence of 3AB at a 30-min period after irradiation of human leucocytes, we performed this study in intracellular NAD levels and PARP activity comparing a group of patients genetically predisposed to colon cancer with a group of healthy volunteers. Our results support our previous results indicating an involvement of PARP in the recovery of DNA strand breaks induced by γ-rays in human leucocytes. In fact, we observed a significant increase ($P \leq 0.01$) in intracellular NAD after 30 min post irradiation in the presence of 3AB (Figure 1A). In the absence of 3AB, the intracellular NAD levels decrease 30 min after irradiation. The direct assay of enzyme activity in permeabilized cells has shown that enzyme activity increases after 30 min post irradiation with a dose of 2 Gy, giving a maximal twofold stimulation of the enzyme activity. This suggests a correlation between the drop in the intracellular NAD and the activity of PARP.

Additionally, in the present study, the PARP activity was shown to be stimulated by the effect of γ-radiation in healthy volunteers but not in FAP patients' leucocytes. Two different methods have been used to assess a potential involvement of PARP on the repair of DNA damage induced by γ-radiation. The results of intracellular NAD levels have shown a significant increase ($P \leq 0.01$) of pyridine nucleotides when we have inhibited the enzyme PARP by 3AB in irradiated and non-irradiated cells from healthy volunteers (Figure 1A and Table 1). These results are consistent with the inhibition of poly(ADP-ribose) synthesis associated with the constitutive form of PARP activity (non-irradiated cells) and with the stimulation of PARP activity by γ-rays. In fact, the effect of γ-radiation results in a significant increase ($P \leq 0.05$) in the PARP activity (Figure 1B). These data agree with the requirement of DNA containing single- or double-strand breaks for the activation of poly(ADP-ribose) synthesis from $NAD^+$ (Benjamin and Gill, 1980).

We did not observe this cellular response to γ-rays in FAP patients' leucocytes. In contrast to normal leucocytes, the inhibition of PARP by 3AB does not increase NAD levels in cells from FAP patients (Figure 1A and Table 1). These results may be related to some defect for $NAD^+$ consumption related to poly(ADP-ribose)polymerase synthesis in DNA repair in FAP patients. Additionally, the metabolism of NAD may be altered in FAP cells as total NAD levels are lower in leucocytes from FAP patients than in cells from healthy individuals.

The estimation of PARP activity in cells from FAP patients has demonstrated that endogenous PARP activity in healthy volunteers and FAP cells is similar. However, we could not observe a stimulation of PARP activity by γ-rays in FAP patients. An association between a deficient PARP response to hydrogen peroxide and conditions with colorectal cancer predisposition has been demonstrated previously in ulcerative colitis and in colorectal adenoma cases (Markowitz et al, 1988).

It seems that the absence of activation of the enzyme by γ-rays in FAP patients may not depend on the frequency of induction of DNA breaks by γ-rays in FAP as our previous work does not reveal any difference in the induction of DNA strand breaks by γ-radiation between healthy individuals and FAP patients (Brás et al, 1995).

Although our results should be regarded as preliminary, the differences observed in the activation of PARP by γ-rays may be important in correlating susceptibility to colon cancer and the involvement of PARP in DNA repair. Several reports indicate a role for defective DNA repair in the aetiology of human cancers (Cleaver, 1994; Kolodner, 1995; Griffin, 1996). Recently, Bièche et al (1996) demonstrated a possible involvement of the PARP gene in the repair of human breast tumour cells.

Further studies on PARP, namely using the Western immunological assay of the amount of poly(ADP-ribose)polymerase protein in the samples, and the study of the PARP gene may help in the correlation and consolidation of our observations concerning the absence of PARP activation by γ-rays observed in FAP and the deficiency in the DNA break repair system contributing to colon cancer progression in familial adenomatous polyposis.

### ABBREVIATIONS

PARP, poly(ADP-ribose)polymerase; NAD, nicotinamide adenine dinucleotide; FAP, familial adenomatous polyposis; APC, adenomatous polyposis coli

### ACKNOWLEDGEMENTS

We thank Professors A Bürkle, RE Pinto, J Mexia, Dr M Sá da Costa, Ms E Ramalho and A Bettencourt who greatly contributed to this work. We extend our appreciation to patients and the healthy volunteers who generously collaborated in this study. L Cristóvão was supported by a doctoral fellowship from the PRAXIS XXI Programme.

### REFERENCES

Almeida R, Fidalgo P, Ramalho E, Brás A, Leitão N, Mira C, Rueff J and Monteiro C (1996*a*) Presymptomatic diagnosis in Portuguese FAP families using intragenic RFLPs and $(CA)_n$ flanking markers by fluorescence based semiautomated DNA analysis. *J Med Genet* **33**: 244–247

Almeida R, Morton N, Fidalgo P, Leitão N, Mira C, Rueff J and Monteiro C (1996*b*) APC intragenic haplotypes in familial adenomatous polyposis. *Clin Genet* **50**: 483–485

Althaus FR and Richter C (1987) ADP-ribosylation of proteins. Enzymology and biological significance. In *Molecular Biology, Biochemistry and Biophysics*, Vol. 37. Springer: Berlin

Benjamin RC and Gill DM (1980) Poly(ADP-ribose) synthesis in vitro programmed by damaged DNA. A comparison of DNA molecules containing different types of strand breaks. *J Biol Chem* **255**: 10502–10508

© Cancer Research Campaign 1998

Bièche I, De Murcia G and Lidereau R (1996) Poly(ADP-ribose) polymerase gene expression status and genomic instability in human breast cancer. *Clin Cancer Res* **2**: 1163–1167

Birnboim HC (1986) DNA strand breaks in human leukocytes induced by superoxide anion, hydrogen peroxide and tumour promoters are repaired slowly compared to breaks induced by ionizing radiation. *Carcinogenesis* **7**: 1511–1517

Brás A, Cristóvão L, Coelho C, Hilali A, Dutrillaux B, Léonard A and Rueff J (1995) Normal genetic response to gamma irradiation in familial adenomatous polyposis. *Eur J Cancer* **31A**: 1506–1510

Bráz J and Lechner MC (1986) ADP-ribosylation of nuclear proteins is increased by phenobarbital: identification of the ADP-ribosylated histone fractions in rat liver nuclei. *FEBS Lett* **199**(2), 164–168

Bürkle A, Müller M, Wolf I and Küpper J-H (1994) Poly(ADP-ribose)polymerase activity in intact or permeabilized leukocytes from mammalian species of different longevity. *Mol Cell Biochem* **138**: 85–90

Chambon P, Weill JD, Doly J, Strosser MT and Mandel P (1966) On the formation of a novel adenylic compound by enzymatic extracts of liver nuclei. *Biochem Biophys Res Commun* **25**: 638–643

Cleaver JE (1994) It was a very good year for DNA repair. *Cell* **76**: 1–4

Cristóvão L and Rueff J (1996) Effect of a poly(ADP-ribose)polymerase inhibitor on DNA breakage and citotoxicity induced by hydrogen peroxide and γ-radiation. *Teratogenesis Carcinogenesis Mutagenesis* **16**: 219–227

Cristóvão L, Rueff J, Brás A, Almeida R, Sanches R, Sá Da Costa M, Mexia J, Silva MJ and Matos L (1993) Radiosensitivity in familial adenomatous polyposis (abstract) *Mutat Res* **291**: 224

de Murcia G, Masson M, Trucco C, Dantzer F, Oliver J, Flatter E, Niedergang C, Ricoul M, Dutrillaux B, Dierich A, Lemeur M, Waltzinger C, Chambon P, Ménissier-de Murcia J (1997) Poly(ADP-ribose) polymerase interacts with the base excision repair factor XRCCI and is required in recovery from DNA damage in mice and in cells. *Mutat Res* **379** (suppl.1): S28

Dukacz BW, Omidiji O, Gray DA and Shall S (1980) (ADP-ribose)$_n$ participates in DNA excision repair. *Nature* **283**: 593–598

Flick K, Schneider R, Auer B, Hirsch-Kauffmann M and Schweiger M (1992) No abnormalities in the NAD(+) ADP-ribosyltransferase(polymerizing) gene of transformed cells from a Fanconi's anaemia patient. *Hum Genet* **89**: 690–691

Gille JJP, Van Berkel CGM, Mullaart E, Vijg J and Joenje H (1989) Effects of lethal exposure to hyperoxia and to hydrogen peroxide on NAD(H) and ATP pools in Chinese hamster ovary cells. *Mutat Res* **214**: 89–96

Gradwohl G, Ménissier-de Murcia J, Molinete M, Simonin F, Koken M, Hoeijmakers JHJ and de Murcia G (1990) The second zinc-finger domain of poly(ADP-ribose)polymerase determines specificity for single-stranded breaks in DNA. *Proc Natl Acad Sci USA* **87**: 2990–2994

Griffin S (1996) DNA damage, DNA repair and disease. *Curr Biol* **6**: 497–499

Grube K and Bürkle A (1992) Poly(ADP-ribose) polymerase activity in mononuclear leukocytes of 13 mammalian species correlates with species-specific life span. *Proc Natl Acad Sci USA* **89**: 11759–11763

Heller B, Wang Z, Wagner EF, Radons J, Bürkle A, Fehsel K, Burkart V and Kolb H (1995) Inactivation of the poly(ADP-ribose) polymerase gene affects oxygen radical and nitric oxide toxicity in islet cells. *J Biol Chem* **270**: 11176–11180

Ikejima M, Noguchi S, Yamashita R, Ogura T, Sugimura T, Gill M and Miwa M (1990) The zinc fingers of human poly(ADP-ribose)polymerase are differentially required for the recognition of DNA breaks and nicks and the consequent enzyme activation. *J Biol Chem* **265**: 21907–21913

Jacobson EL and Jacobson MK (1976) Pyridine nucleotide levels as a function of growth in normal and transformed 3T3 cells. *Arch Biochem Biophys* **175**: 627–634

Kolodner RD (1995) Mismatch repair: mechanisms and relationship to cancer susceptibility. *Trends Biochem Sci* **20**: 397–401

Lechner MC and Bráz J (1985) Nuclear ADP-ribosyl transferase activity correlates with induction of P-450 monooxygenases by phenobarbital in rat liver microsomes. *Eur J Biochem* **151**: 621–624

Lindahl T, Satoh MS and Dianov G (1995*a*) Enzymes acting at strand interruptions in DNA. *Phil Trans R Soc Lond B Biol Sci* **347**: 57–62

Lindahl T, Satoh T, Poirier GG and Klungland A (1995*b*). Post-translational modification of poly(ADP-ribose)polymerase induced by DNA strand breaks. *Trends Biochem Sci* **20**: 405–411

Markowitz MM, Rozen P, Pero RW, Tobi M and Miller DG (1988) Hydrogen peroxide induced adenosine diphosphate ribosyl transferase (ADPRT) response in patients with inflammatory bowel disease. *Gut* **29**: 1680–1686

Molinete M, Vermeulen W, Bürkle A, Ménissier-de Murcia J, Heiner Küpper J, Hoeijmakers JHJ and de Murcia G (1993) Overproduction of the poly(ADP-ribose) polymerase DNA-binding domain blocks alkylation-induced DNA repair synthesis in mammalian cells. *EMBO J* **12**: 2109–2117

Murnane JP (1996) Role of induced genetic instability in the mutagenic effects of chemicals and radiation. *Mutat Res* **367**: 11–23

Nishizuka Y, Ueda K, Honjo T and Hayaishi O (1968) Enzymic adenosine diphosphate ribosylation of histone and poly adenosine diphosphate ribose synthesis in rat liver nuclei. *J Biol Chem* **243**: 3765–3767

Panzeter PL and Althaus FR (1994) DNA strand break-mediated partitioning of poly(ADP-ribose) polymerase function. *Biochemistry* **33**: 9600–9605

Sancar A (1995) DNA repair in humans. *Annu Rev Genet* **29**: 69–105

Satoh MS and Lindahl T (1992) Role of poly(ADP-ribose) formation in DNA repair. *Nature* **356**: 356–358

Satoh MS and Lindahl T (1994) Enzymatic repair of oxidative DNA damage. *Cancer Res* **54** (suppl.): 1899s–1901s

Satoh MS, Poirier GG and Lindahl T (1993). $NAD^+$-dependent repair of damaged DNA by human cell extracts. *J Biol Chem* **268**: 5480–5487

Satoh MS, Poirier GG and Lindahl T (1994) Dual function for poly(ADP-ribose)synthesis in response to DNA strand breakage. *Biochemistry* **33**: 7099–7106

Schweiger M, Auer B, Burtscher HJ, Hirsch-Kauffmann M, Klocker H and Schneider R (1987) DNA repair in human cells: biochemistry of the hereditary diseases Fanconi's anaemia and Cockayne syndrome. *Eur J Biochem* **165**: 235–242

Skidmore CJ, Davies MI, Goodwin PM, Halldorsson H, Lewis PJ, Shall S and Zia'ee A-A (1979) The involvement of poly(ADP-ribose)polymerase in the degradation of NAD caused by γ-radiation and N-methyl-N-nitrosourea. *Eur J Biochem* **101**: 135–142

© Cancer Research Campaign 1998